# A Nematode of the Mid-Atlantic Ridge Hydrothermal Vents Harbors a Possible Symbiotic Relationship

**DOI:** 10.3389/fmicb.2018.02246

**Published:** 2018-09-20

**Authors:** Laure Bellec, Marie-Anne Cambon-Bonavita, Valérie Cueff-Gauchard, Lucile Durand, Nicolas Gayet, Daniela Zeppilli

**Affiliations:** ^1^IFREMER, Centre Brest, REM/EEP/LEP, ZI de la pointe du diable, CS10070, Plouzané, France; ^2^IFREMER, Centre Brest, UMR 6197 – Laboratoire de Microbiologie des Environnements Extrêmes (REM/EEP/LM2E), ZI de la pointe du diable, CS10070, Plouzané, France; ^3^CNRS, UMR 6197 – Laboratoire de Microbiologie des Environnements Extrêmes (LM2E), Institut Universitaire Européen de la Mer (IUEM), Technopole Brest-Iroise, Plouzané, France; ^4^Université Bretagne Occidentale (UBO), UMR 6197 – Laboratoire de Microbiologie des Environnements Extrêmes (LM2E), Institut Universitaire Européen de la Mer (IUEM), Technopole Brest-Iroise, Plouzané, France

**Keywords:** sulfur-oxidizing bacteria, Lucky Strike vent field, meiofauna, nematode, endosymbiont

## Abstract

Deep-sea hydrothermal vent meiofauna have been the focus of recent research and the discovery of an abundant well-adapted free-living marine nematode on the Mid-Atlantic Ridge offers new perspectives on adaptations to the vent environment. Indeed, knowledge concerning biological interactions of microbes and meiofauna in marine extreme environments is scarce, especially for nematodes. In this study, we used microscopic observations [fluorescence *in situ* hybridization (FISH) and scanning electron microscopy (SEM)] and metabarcoding of 16S rRNA to characterize the bacterial community of the nematode species *Oncholaimus dyvae*, an overlooked but ecologically important vent organism. Detection of bacteria in the buccal cavity and on the cuticle (SEM) and epibionts in its intestine (FISH) suggests that *O. dyvae* harbors its own bacterial community. Molecular results and phylogenetic analysis show that bacteria associated with this species are related to symbiotic lineages typical of hydrothermal vent fauna, such as sulfur-oxidizing bacteria related to *Epsilonproteobacteria* and *Gammaproteobacteria*. This multi-approach study suggests a potential symbiotic role of bacteria with its nematode host and opens new research perspectives on vent meiofauna.

## Introduction

Deep-sea hydrothermal vents are dynamic and ephemeral ecosystems located on mid-ocean ridges or back-arc basins associated with volcanic, tectonic and magmatic processes (Van Dover, 2000). These extreme environments are hot spots of productivity and biomass compared to other deep-sea environments, harboring organisms well adapted to high concentrations of reduced compounds. Among the most studied hydrothermal vents are those in the Lucky Strike (LS) field, discovered during the FAZAR French-American cruise in 1992 (Langmuir et al., 1993; Wilson et al., 1996). This Mid-Atlantic Ridge area extends over one square kilometer at a mean depth of 1700 m and contains more than 20 sulfide edifices distributed around a central lava lake (Ondréas et al., 2009). LS fauna assemblages are dominated by mussel beds (*Bathymodiolus azoricus*), and shrimp populations (*Mirocaris fortunata*) (Desbruyères et al., 2001). A recent study (Husson et al., 2017) focusing on assemblages of *B. azoricus* confirmed the dominant position of this species, which constitutes nearly 90% of the biomass (in g dry weight/m^2^) of this ecosystem. This species lives in symbiosis with both methane- and sulfur-oxidizing *Gammaproteobacteria* located in their gill epithelial cells (Duperron et al., 2006). Symbioses, specifically chemosynthetic symbioses which occur between bacteria and marine invertebrates were discovered only 40 years ago, at the Galapagos hydrothermal vents, where the gutless tube worm *Riftia pachyptila* was first observed. It has since become apparent that chemosynthetic symbioses are present in a wide range of habitats from shallow-water coastal sediments to deep-sea environments, such as hydrothermal vents (Dubilier et al., 2008). On the LS site, *B. azoricus* harbors endosymbionts that are fuelled by reduced compounds emitted by hydrothermal vent fluids, which contribute partially to their host’s nutrition (Cavanaugh et al., 1981; Van Dover, 2000; Van Dover et al., 2002). It seems that the ratio between densities of methane- and sulfur-oxidizers, or their gene expression could fluctuate in response to the environmental conditions, but the molecular mechanisms of regulation between *Bathymodiolus* and its symbionts are still unclear (Bettencourt et al., 2014; Szafranski et al., 2015). A study using 16S rRNA sequence analysis and fluorescence *in situ* hybridization (FISH) showed a specific association between *Bathymodiolus* mussels and a new family of *Epsilonproteobacteria* (Assié et al., 2016). Hundreds of animal species from different phyla are now known to live in chemosynthetic symbiosis and, with the improvement of molecular methods, the discovery of many more is expected, especially among smaller species.

Meiofaunal organisms, small benthic invertebrates living in aquatic systems, are important contributors to ecosystem processes and functions due to their high abundance, diversity, widespread distribution, rapid generation times and high metabolic rates (Zeppilli et al., 2015a). Several studies have shown that meiofauna can adapt to extreme environments, including hydrothermal vents. Deep-sea hydrothermal vent meiofauna are a recent focus of research attracting growing interest, as meiofauna may represent up to 20% of the total faunal diversity of these ecosystems (Zeppilli et al., 2017). Only few studies including meiofauna have been conducted on the Mid-Atlantic Ridge but these show that meiofaunal organisms (mainly composed of generalist nematodes and endemic copepods) contribute up to 50% of the total faunal diversity (Zekely et al., 2006; Sarrazin et al., 2015). Moreover, hydrothermal activity and local environmental conditions influence the composition and distribution of nematode or copepod communities (Cuvelier et al., 2014; Zeppilli et al., 2015b; Plum et al., 2017). One very well known shallow hydrothermal vent nematode is *Oncholaimus campylocercoides* (Gerlach and Riemann, 1973; Thiermann et al., 2000). This species can tolerate extreme temperatures and high sulfide concentrations (Gerlach and Riemann, 1973; Thiermann et al., 2000). The genus was reported for the first time at the LS hydrothermal vent field where it is present at high abundance (Tchesunov et al., 2012; Zeppilli et al., 2015b). A new species named *Oncholaimus dyvae* was recently fully described, with related data on abundance, biomass, diet, phylogeny, microscopic observations (SEM) and a detailed morphological description (Zeppilli et al., unpublished). High densities of *O. dyvae* (subfamily Oncholaiminae) were reported in association with *Bathymodiolus* assemblages at Eiffel Tower, at Cypress and Y3 hydrothermal vent sites (LS hydrothermal vent field) and also in the organic colonization substrata deployed around the Eiffel Tower (Zeppilli et al., 2015a; unpublished). This species was only reported near active vent emissions and it was absent in the inactive vents and surrounding sediments (Zeppilli et al., unpublished). Carbon isotopic analyses showed that *O. dyvae* is a detritivore/bacterivore, consuming both thiotrophic and methanotrophic producers while no direct trophic link between the nematode *O. dyvae* and its bivalve host was detected (Zeppilli et al., unpublished). The byssus could be a shelter, as well as a trap for organic matter or simply a physically suitable three-dimensional substratum for *O. dyvae* (Zeppilli et al., unpublished).

The discovery of a new abundant species of nematode living in association with a well-known symbiotic host (*B. azoricus*) under the same extreme environment raises new scientific questions: Does *O. dyvae* have its own bacterial community? Is it possible to characterize it? Are we in presence of two clearly independent associations (bacteria – mussel and bacteria – nematode) or a tripartite system (mussel – nematode – bacteria)? The aim of this study was to identify and characterize a possible bacterial community of a neglected but important vent organism. Our approach was based on microscopic observations (SEM and FISH) coupled with molecular diversity assessment using metabarcoding based on the 16S rRNA gene.

## Materials and Methods

### Study Area and Sample Collection

The LS hydrothermal field is located on the Mid-Atlantic Ridge, south of the Azores (**Supplementary Figure S1**). It is a large vent field at a mean depth of 1700 m, with a central lava lake of ∼200 m diameter, surrounded by 3 volcanic cones and more than 20 sulfide edifices (Ondréas et al., 2009). The vent field and hydrothermal fluids occur in four main areas. This study focuses on the southeastern area where many vent edifices are being studied, such as the *Eiffel Tower* or *Montsegur*.

Samples were collected during three oceanographic cruises on the research vessel “*Pourquoi pas?*”: Biobaz (2013), Momarsat (2014), and Momarsat (2017). Sample collection was carried out using the remotely operated vehicle (ROV) Victor6000. Samples were obtained from a broad-scale study on the structure of *B. azoricus* assemblages at LS initiated by Sarrazin et al., (2012, unpublished). These assemblages were collected from the Eiffel Tower edifice (37°17.34′N, 32°16.53′W) during the Biobaz (2013) and Momarsat (2014) cruises and Montsegur edifice (37°17.28′N, 32°16.54′W) during the Momarsat 2017 cruise. The fauna was sampled using the suction sampler of the ROV and arm grab, following the protocol described in Cuvelier et al. (2011).

### Nematode Sorting and Fixation

Nematodes were sorted under a stereomicroscope (M125; Leica, Wetzlar, Germany) from the mussel assemblages, which more exactly meant separating them from the byssus of *B. azoricus*. We selected one of the most abundant species, *O. dyvae*, a large nematode (> 8 mm) distinguishable from the other species by its morphological characteristics (Zeppilli et al., unpublished). A set of specimens was immediately frozen at –80°C for later molecular analyses (on both nematodes and microbial diversity). Other specimens were stored for Scanning Electron Microscopy (SEM) studies: these nematodes were fixed in glutaraldehyde 2.5% for 16 h at 4°C (Eisenback, 1985), then transferred to a sodium azide solution (0.065 g in 150 ml filtered sea-water) and stored at 4°C until use. Another set of nematodes was stored for FISH analyses: samples were fixed for 2 h in 3% formaldehyde-sterile seawater solution and rinsed with 1X phosphate-buffered saline (PBS)–sterile seawater solution (1:1). These samples were stored in absolute ethanol- 2X PBS solution (1:1) at -20°C until use (Durand et al., 2010).

### Nematode DNA Extraction, PCR, and Sequencing

The nematodes directly frozen at -80°C from Momarsat, 2017 were identified using a molecular approach. Total DNA was extracted from each whole nematode individually, using the Qiagen^®^ DNeasy Blood & Tissue kit following manufacturer’s instructions. Additionally, one negative extraction control was performed. A partial fragment of the 28S rRNA gene (654 bp; the large subunit of ribosomal DNA) was amplified with the primer sets D2Ab (5′-ACAAGTACCGTGAGGGAAAGTTG-3′) and D3B (5′-TCGGAAGGAACCAGCTACTA-3′) (De Ley et al., 1999). PCRs were performed on a Gene-Amp^TM^ PCR system 9700 thermocycler (Applied Biosystems, Forster City, CA, United States) in a final volume of 25 μl using the following mix: 2 μl of extracted DNA were added to 5 μl 5X PCR buffer, 5 mM of each dNTP, 50 mM MgCl_2_, 20 μM of each of the two primers and 0.1 μl Taq polymerase (5U/μl – Promega). The PCR cycles were 2 min at 94°C then 30 cycles of 30 s denaturation at 94°C, 45 s annealing at 55°C and 2 min extension at 72°C, followed by 10 min at 72°C for final elongation. All amplification products were run on a 0.8% agarose-TAE gel to verify the size of the amplicons. Purification and Sanger sequencing of PCR products were performed by Macrogen^1^. Chromatograms were assembled and edited using Geneious 8.1.9^2^ (Kearse et al., 2012) and all nucleotide differences were checked visually.

### 16S rRNA Bacterial Genes

DNA from 12 *O. dyvae* (after morphological and genetic identification) collected during the Momarsat, 2017 cruise were sent to MR DNA^3^ (Shallowater, TX, United States) for amplification of prokaryotic diversity. Sequencing was performed on a 450 bp fragment of the 16S rRNA gene (16S) using Illumina’s MiSeq technology. Briefly, the 16S V3-V4 variable region (Fadrosh et al., 2014) (341/785 primers, with barcode on the forward primer) was used in a 28-cycle PCR using the HotStarTaq Plus Master Mix Kit (Qiagen, United States) under the following conditions: 94°C for 3 min, followed by 28 cycles of 94°C for 30 s, 53°C for 40 s and 72°C for 1 min, after which a final elongation step was performed at 72°C for 5 min. Multiple individual nematodes were pooled together in equal proportions based on their molecular weight and DNA concentrations. Pooled samples were purified using calibrated Ampure XP beads. The pooled and purified PCR product was used to prepare a DNA library by following the Illumina TruSeq DNA library preparation protocol. Sequencing was performed at MR DNA on a MiSeq sequencer using the 2 × 300 bp chemistry.

### Bioinformatic Data Processing

Prokaryotic 16S rRNA paired-end reads were merged using USEARCH (Edgar and Flyvbjerg, 2015) after q25 trimming of the ends. The resulting 16S reads were processed using the Find Rapidly OTU with Galaxy Solution (FROGS) v2 pipeline (Escudié et al., 2018). In short, sequences were depleted of barcode, then sequences < 300 bp and those containing an ambiguous base were removed. Next, reads were clustered into *de novo* operational taxonomic units (OTUs) using Swarm (Mahé et al., 2014) with an aggregation distance equal to 3. Chimera were then removed with VSEARCH (Rognes et al., 2016). Additionally, filters were applied to the OTUs: one for abundance, with an optimal threshold of 0.005% (Bokulich et al., 2013), and one for OTU occurrence (sequences had to be present in 5 samples). The OTUs finally selected were taxonomically assigned by BLASTn + (Camacho et al., 2009) using the Silva release 128 reference database (Quast et al., 2012). Finally, filtrations on BLAST taxonomic affiliation were performed, with a minimum coverage of 95% and a minimum identity of 60%. OTU structure, visualization, composition analysis and alpha diversity indices were performed using phyloseq (McMurdie and Holmes, 2013) available through FROGS. Parts of the visualization were also done with the Phinch program (Bik and Interactive, 2014). The 16S rRNA data are available in the NCBI SRA repository (Accession SRP156406, BioProject PRJNA484672).

### Scanning Electron Microscopy (SEM) Observations

Six *Oncholaimus dyvae* specimens were used for SEM observations to check for the presence of prokaryotes on the external cuticle of the animals. After fixation on board the research vessel (see *Nematode sorting and fixation*), nematodes were postfixed in 0.8% osmium tetroxide for 20 h at 4°C and then dehydrated by ethanol series. Nematodes were desiccated using a critical-point dryer (CPD 300; Leica, Wetzlar, Germany) and then mounted on a specimen stub. They were gold-coated using an SCD 040 (Blazers Union, Blazers, Liechtenstein). Observations were made with a Quanta 200 MK2 microscope (FEI, Hillsboro, OR, United States) and xT microscope software (FEI).

### Fluorescence *in situ* Hybridization

We performed FISH to identify the occurrence of prokaryotes on nine nematodes collected during the Momarsat, 2017 cruise. On board ship, some nematodes were fixed (see *Nematode sorting and fixation*). Later, at the laboratory, these specimens were hybridized with universal probes (Eurogentec, Liège, Belgium) (**Table 1**). Whole nematodes were rinsed in a 30 or 40% formamide-buffer. Then, they were incubated in a final volume of 30 μl of a hybridization buffer containing 30 or 40% formamide and 3 μl of each probe at 8 μM for 3.5 h at 46°C. The nematodes were then rinsed in a washing buffer for 45 min at 48°C. This step was ended by a final wash in milliQ water at room temperature for 10 min. After a quick drying period, the labeled organisms were mounted on a slide in an anti-fade mounting medium (SlowFade^®^ Gold anti-fade reagent, Invitrogen) containing DAPI (4′,6-diamidino-2-phenylindole, dilactate), a DNA intercalary agent. Observations were made on an Imager.Z2 microscope (Zeiss, Oberkochen, Germany) equipped with a slider module Apo-Tome (Zeiss) and Colibri light technology (Zeiss) and using an AxioCam MRm (Zeiss) camera. Micrographs were analyzed using Zen (Zeiss) software.

**Table 1:** Fluorescent probes used in this study.

Phylotype	Probe	Probe sequence (5′-3′)	Position (rRNA genes)	Formamide (%)	Reference
*Eubacteria*	Eub338	GCTGCCTCCCGTAGGAGT	338 (16S)	30–40	Amann et al., 1990
*Deltaproteobacteria*	Delta495a	AGTTAGCCGGTGCTTCCT	495 (16S)	30	Loy et al., 2002
*Epsilonproteobacteria*	EPSY549	CAGTGATTCCGAGTAACG	549 (16S)	30–40	Lin et al., 2006
*Gammaproteobacteria*	GAM42a	GCCTTCCCACATCGTTT	1027 (23S)	30–40	Manz et al., 1992
Nonsense	Non338	ACTCCTACGGGAGGCAGC	338 (16S)	30	Wallner et al., 1993


### Phylogenetic Reconstruction

Epsilonproteobacterium-related sequences plus one outgroup (*Thiomicrospira halophile*) were used in the analysis. A dataset of the 16S rRNA gene was aligned with MUSCLE alignment implemented in Geneious 8.1.9 (Kearse et al., 2012) and then processed in Gblocks (version 0.91b) in order to remove gaps (397 bp final length). Phylogenetic reconstruction was performed using Mr Bayes 3.2.6 (Ronquist et al., 2012) on the CIPRES Science Gateway (Miller et al., 2010). The best-fitting model of evolution was computed with jmodeltest2 (Darriba et al., 2012). Bayesian analysis was carried out with 4 chains of 30 × 10^6^ generations, with trees sampled every 1000 generations, and the burn-in value set at 25% of the sampled trees. We checked that the standard deviation of the split frequencies fell below 0.01 and confirmed convergences of the runs to ensure convergence in the tree search using Tracer v1.6 software^4^. The tree was visualized using FigTree v1.4.3^5^. *Epsilonproteobacteria* sequences are available in GenBank with accession numbers from MH458848 to MH458855.

## Results

First, we identified (morphological and 28S rRNA analyses) all the nematode specimens recovered from LS and used in this study as *O. dyvae* (Zeppilli et al., unpublished).

### SEM Observations on Bacteria

**FIGURE 1:**
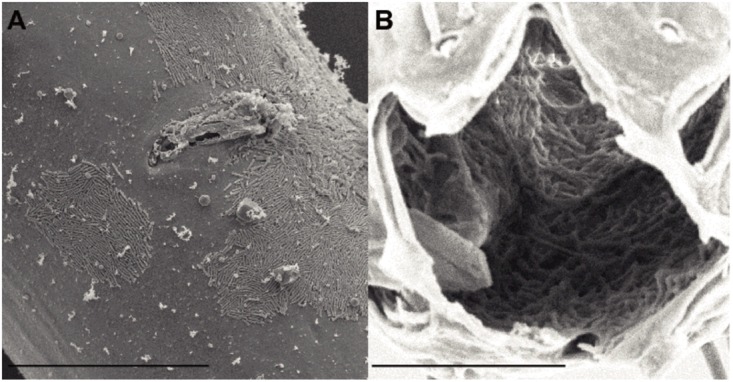
Scanning electron micrographs of *Oncholaimus dyvae* **(A)** vulval region, ventral view (female) **(B)** Buccal cavity. Scale bars **(A)** 30 μm, **(B)** 10 μm

Scanning electron microscopy observations of six specimens revealed the presence of bacteria, which mainly had a rod-shaped morphology, located on the cuticle and in the buccal cavity (**Figure 1**). All nematodes examined, three from Biobaz (2013) and three from Momarsat (2014), harbored rod-shaped bacteria in their mouth cavity (**Table 2**). Intact microbial mats were observed on four individuals: one from the Biobaz (2013) cruise and three from the Momarsat (2014) cruise. These mats were observed on the posterior third of the nematode body and seemed to show two different morphotypes of bacteria (rod-shaped or coccoid).

**Table 2:** List of SEM observations.

Cruise		Sex	Location in the body			
			
			Buccal cavity		Posterior part	
				
			Prokaryotes	Type	Prokaryotes	Type
Biobaz, 2013		F	Yes	Rod-shaped	No	
Biobaz, 2013		M	Yes	Rod-shaped	Yes	Rod-shaped
Biobaz, 2013		M	Yes	Rod-shaped	No	
Momarsat, 2014		F	Yes	Rod-shaped	Yes	Coccoid
Momarsat, 2014		F	Yes	Rod-shaped	Yes	Rod-shaped
Momarsa, 2014		M	Yes	Rod-shaped	Yes	Coccoid


### FISH Observations on Bacteria

**FIGURE 2:**
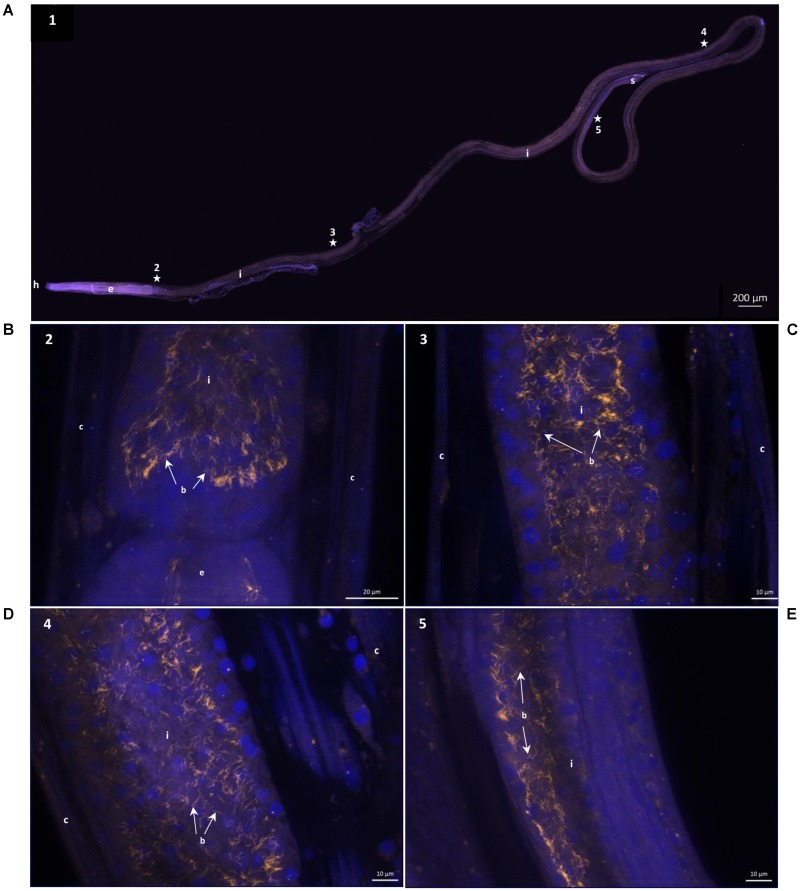
16S rRNA Fluorescence *in situ* hybridization of *Oncholaimus dyvae*. **(A)** Whole male *O. dyvae*. **(B–E)** Long thin *Epsilonproteobacteria* throughout the intestine of the host. The position of each image on the whole specimen is shown by a numbered star. In blue, DAPI-stained host nuclei; in orange, *Epsilonproteobacteria* hybridized with a Cy3-labeled probe. b = bacteria, c = cuticle, e = esophagus, h = head, i = intestine, s = spicules.

Nine specimens of *O. dyvae* from the Momarsat (2017) cruise were analyzed to reveal the occurrence of bacteria. Individuals observed using FISH showed long, thin bacteria inside their buccal cavity, esophagus and intestine (**Figure 2**). The photographs show the nuclei of eukaryotic cells labeled with DAPI (blue) along with a fluorescent orange signal corresponding to specific hybridization of the bacterial probe labeled with Cy3. We obtained positive results with a general bacterial probe (photographs not shown), and with the *Epsilonproteobacteria* probe (**Figure 2**). General bacterial probe and *Epsilonproteobacteria* probe do not show different morphologies, they co-hybridize. We observed no fluorescence with *Gammaproteobacteria* or *Deltaproteobacteria* probes. We checked for autofluorescence and absence of signal using non-hybridized specimens and a nonsense probe (negative controls). These results showed intact and active *Epsilonproteobacteria* (long thin filaments) with a high fluorescent signal, but not inside the cells of the hosts, suggesting epibiotic bacteria in the intestine.

### Bacterial Communities of *O. dyvae*

**FIGURE 3:**
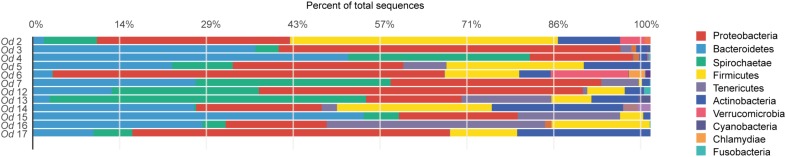
Bacterial community distribution at the phylum level in 12 *Oncholaimus dyvae*. Relative abundance is represented in terms of percentage of the total effective bacterial sequences per specimen.

Our metabarcoding (region V3V4 of the 16S rRNA gene) of the bacterial communities associated with 12 *O. dyvae* specimens produced a total of 768,074 sequence reads (**Supplementary Table S1**). The reads were filtered, resulting in 522,783 sequences clustered in 223 OTUs, taxonomically assigned with the Silva database (**Supplementary Table S2**). Considering OTUs from all specimens, the bacterial community composition of *O. dyvae* at LS was dominated by *Proteobacteria*-related sequences, which represented 33% of total reads and a diversity of 113 OTUs, followed by *Bacteroidetes* (25%), *Spirochaetae* (16%) and *Firmicutes* (9%) (**Figure 3**). While there were overall similarities across individuals, we also observed variability in the relative abundance of OTUs, as six nematodes harbored a majority of *Proteobacteria* (*Od* 3, *Od* 5, *Od* 6, *Od* 7, *Od* 12, and *Od* 17), four a majority of *Bacteroidetes* (*Od* 4, *Od* 14, *Od* 15, and *Od* 16), one a majority of *Spirochaetae* (*Od* 13) and one of *Firmicutes* (*Od* 2). The bacterial composition of some nematodes was clearly dominated by one phylum, for example *Proteobacteria* (*Od* 6, *Od* 17) whereas other specimens (*Od* 5, *Od* 7) harbored a more even composition of phyla. *Proteobacteria* and *Bacteroidetes* were present in all nematodes, forming between 16 to 63 and 2 to 54 of total reads per nematode, respectively. The two other major phyla (*Spirochaetae* and *Firmicutes*) were almost absent and constituted fewer than 1% of reads in some nematodes (**Supplementary Table S3**).

**FIGURE 4:**
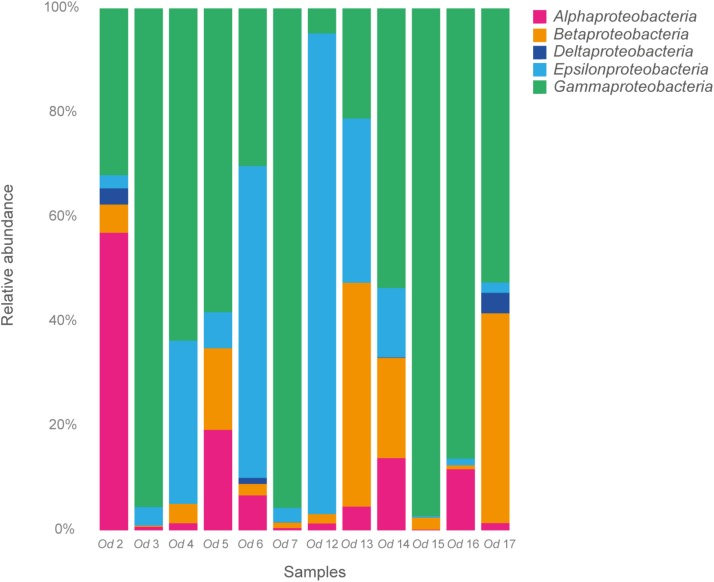
Relative abundance of Proteobacteria in 12 *Oncholaimus dyvae*.

Among the *Proteobacteria*, the *O. dyvae* bacterial community was dominated by sequences related to *Gammaproteobacteria*, which represented 60% of the total sequences, then *Epsilonproteobacteria* (24%), *Betaproteobacteria* (8%), *Alphaproteobacteria* (7%) and finally *Deltaproteobacteria* (1%) (**Figure 4**). However, the structure of these communities was slightly different, as eight nematodes harbored a majority of *Gammaproteobacteria*, two a majority of *Epsilonproteobacteria*, one a majority of *Alphaproteobacteria* and one mostly *Betaproteobacteria*. These four classes of *Proteobacteria* were present in all *O. dyvae* samples, with different relative abundances, but *Deltaproteobacteria* was detected in only three nematodes (*Od* 2, *Od* 6, and *Od* 7). Three nematodes (*Od* 3, *Od* 7, and *Od* 15) harbored an important majority of *Gammaproteobacteria* (> 95%) and one (*Od* 12) was heavily dominated by *Epsilonproteobacteria* (92%), whereas other nematodes (*Od* 13 and *Od* 14) showed a more even distribution of Proteobacterial classes.

**FIGURE 5:**
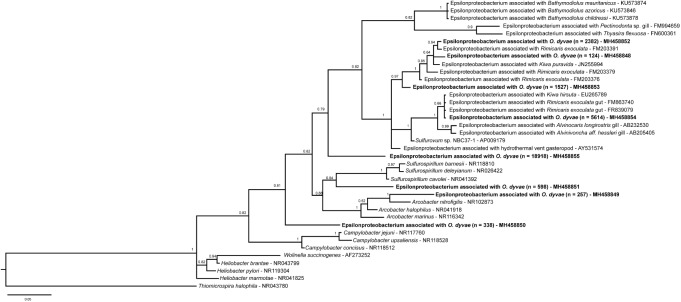
Bayesian inference tree of the 16S rRNA gene for *Epsilonproteobacteria*. Analysis based on the partial sequence (397 bp) and performed under a General Time Reversible model with Gamma-distributed rates of evolution and a proportion of invariant sites with 30 million generations. The numbers are the posterior probabilities reflecting clade support. Representative sequence names in bold are from this study and n is the total number of sequences across all specimens.

Among the *Epsilonproteobacteria*, the cluster analysis revealed 21 OTUs split among five genera (*Arcobacter*, *Campylobacter*, *Sulfurimonas*, *Sulfurospirillum*, and *Sulfurovum*) (**Supplementary Figure S2**). *Sulfurovum* and *Sulfurimonas* sequences were found in all nematodes and represented 97% of total *Epsilonproteobacteria*. The three other genera were detected only in one bacterial community (*Od* 6). A phylogenetic reconstruction performed with representative sequences of the 21 OTUs plus published sequences affiliated to *Epsilonproteobacteria* showed a broad distribution of the *O. dyvae* epsilon related bacterial communities (**Figure 5**). The representative sequence are unique OTUs recovered for the genera [*Arcobacter* (MH458849), *Campylobacter* (MH458850) and *Sulfurospirillum* (MH458851)] and the most abundant OTUs of *Sulfurimonas* (MH 458855) and *Sulfurovum* (MH 458854), the latter two being present in all nematodes. We also add three OTUs of *Sulfurovum* that are slightly different from each other (4–5% of dissimilarity). First, all 16S rRNA sequences from *O. dyvae* inserted into an Epsilonproteobacterium tree, which confirmed BLAST affiliations and grouped within specific clades such as *Arcobacter* or *Sulfurospirillum*. More interestingly, no *Epsilonproteobacteria* sequence from *O. dyvae* belonged to the Epsilonproteobacterium clade associated with *Bathymodiolus* mussels. Most of the new sequences formed a large clade with sequences from hydrothermal organisms such as shrimps (*Rimicaris exoculata* and *Alvinocaris longirostris*), the “yeti crab” *Kiwa*, or snails (*Alviniconcha aff. hessleri*). A representative sequence of an abundant OTU (total number across all specimens = 5614) clustered with sequences from a gut symbiont of *Rimicaris exoculata*, and another representative sequence of *O. dyvae* (total number across all specimens = 2382) clustered with a gill chamber symbiont of *Rimicaris exoculata*.

The *Gammaproteobacteria* diversity was larger than that of *Epsilonproteobacteria*, showing 41 OTUs dominated by the family *Pseudomonadaceae* (11 OTUs of the genus *Pseudomonas*), which was present in all nematodes examined (**Supplementary Figure S2**). This class of bacteria was dominated in terms of number of reads by only one OTU affiliated (93% of identity) with uncultured environmental samples and present across all specimens. Two OTUs (*Methylococcales* and SUP05 cluster) were relevant as their BLAST affiliation matched (100% of identity), respectively with a methanotrophic and a thiotrophic endosymbiont of *B. azoricus* from LS. The methanotrophic OTU represent 349 reads (0.33% of total *Gammaproteobacteria*) mainly from one nematode, *Od* 5, (5% of *Gammaproteobacteria* from *Od* 5). Whereas the thiotrophic OTU was present in all specimens for a total of 1830 reads (1.8% of total *Gammaproteobacteria*). Another example was a Thiotrichaceae OTU with a BLAST assignation (98% of identity) within a *Gammaproteobacteria* endosymbiont of *Alviniconcha* sp.

Alpha diversity indices values for each *O. dyvae* specimen are shown in **Table 3**. We observed that for many bacterial communities, their richness (number of observed OTUs) and Chao1 (richness + estimated number of unobserved OTUs) were similar, suggesting that almost all OTUs were detected. More informatively, samples such as *Od* 12, *Od* 16, and *Od* 17 had the same numbers of OTUs but different Shannon index values. A high Shannon index indicates a high evenness and low value (*Od* 12 and *Od* 16) indicates a low evenness (i.e., a high dominance of a few OTUs) of the bacterial distribution. Indeed, the bacterial community abundances of *Od* 12 or *Od* 16 were dominated by one OTU related to *Epsilonproteobacteria*, *Sulfurimonas* at 58%, and one OTU related to *Mollicutes*, *Spiroplasma* at 35%, respectively, whereas *Od* 17 was composed of many OTUs with few sequences.

**Table 3:** Alpha diversity indices, OTU numbers, species richness (Chao1 and standard error), Shannon indices.

Sample ID	OTU	Chao1 ± SE	Shannon
*Od* 2	117	127 ± 5	2.94
*Od* 3	122	138 ± 8	1.6
*Od* 4	92	102 ± 6	1.54
*Od* 5	118	132 ± 7	3.4
*Od* 6	163	180 ± 8	3.52
*Od* 7	110	168 ± 26	1.63
*Od* 12	101	115 ± 8	2.01
*Od* 13	101	152 ± 26	2.12
*Od* 14	139	149 ± 6	3.6
*Od* 15	82	118 ± 18	1.45
*Od* 16	111	144 ± 15	2.15
*Od* 17	115	159 ± 19	3.09


### Negative Control of Metabarcoding

An analysis of the 12 *O. dyvae* specimens plus a blank (i.e., a negative DNA extraction control) was also performed under FROGS. After bioinformatics processing, the bacterial community associated with this dataset produced 409,303 sequences clustered in 359 OTUs. The blank bacterial community harbored no sequences related to *Epsilonproteobacteria* or *Deltaproteobacteria*. A selection of OTUs (> 1% of the relative abundance of total reads) showed that there were only eight OTUs in common between the blank and the nematodes. Affiliation and relative abundance of OTUs are given in **Supplementary Table S4**. The blank and the nematodes shared bacteria related to *Staphylococcus*, *Streptococcus* or *Pseudomonas*. They did not share methanotrophic or thiotrophic endosymbionts of *B. azoricus* nor sequences close to *R. exoculata*.

## Discussion

### Association Between *O. dyvae* and Bacteria at a Hydrothermal Vent

In this study we found evidence that *O. dyvae* could harbor its own chemoautotrophic bacterial community, including SEM and FISH observations and metabarcoding of the 16S rRNA gene. Microscopic observations made it possible to distinguish intact rod-shaped bacterial forms in the digestive cavity. Active *Epsilonproteobacteria* were found along the entire length of the digestive tract and could be epibionts playing a role in digestion. Metabarcoding results suggested that *O. dyvae* harbors its own bacterial community, which is slightly different from their surrounding habitat (*B. azoricus*). The detection of *Gammaproteobacteria* differed according to the method used: they were detected with amplicon sequencing in all specimens but not by FISH. These results could be due to: (i) *Gammaproteobacteria* being too small for detection using the FISH method; (ii) the probe having a different stringency with *O. dyvae* bacterial associates than with those present in other vent fauna; (iii) most of the *Gammaproteobacteria* consisting of components of the nematode’s diet, which were already digested.

Representatives of symbiotic lineages typical of hydrothermal vent fauna were detected, such as some sulfur-oxidizing symbionts affiliated to *Epsilonproteobacteria* and *Gammaproteobacteria* (Dubilier et al., 2008). *Epsilonproteobacteria* described as ecto- or endosymbionts are harbored by shrimps, crabs, gastropods, polychaetes and mussels and are widespread at hydrothermal vents (Dubilier et al., 2008; Assié et al., 2016). The *Epsilonproteobacterium*-related community associated with *O. dyvae* covered a large diversity with five genera, but was dominated by *Epsilonproteobacteria* close to the epibiotic symbiont of *Rimicaris exoculata*. *Rimicaris* shrimps are well-studied marine organisms that dominate the megafauna at several Mid-Atlantic Ridge sites but not presently at LS. Two distinct chemoautotrophic ectosymbiotic bacterial communities have been described for *R. exoculata*. The first community is a complex one located in the cephalothorax and composed of lineages related to *Epsilon*, *Gamma*, *Zeta*, and *Deltaproteobacteria* (Polz and Cavanaugh, 1995; Zbinden et al., 2004, 2008; Petersen et al., 2010; Hügler et al., 2011; Jan et al., 2014). These bacteria are known as sulfur-, iron- hydrogen and methane-oxidizers that play a role in the nutrition of their host (Ponsard et al., 2013). The second one, in the gut and stomach is composed of lineages related to *Deferribacteres*, Mollicutes, *Epsilon*, and *Gammaproteobacteria* (Zbinden and Cambon-Bonavita, 2003; Durand et al., 2010, 2015). Bacteria from the gut remained after starvation periods, suggesting there was a resident microbial community and proposed to be a symbiotic community (Durand et al., 2010). Sequences from *O. dyvae* were closer to *Epsilonproteobacteria* from the gut or the cephalothorax of *R. exoculata* than to sequences from *B. azoricus* symbionts, while sharing the same habitat. The discovery of an association between deep-sea *Bathymodiolus* mussels and a family of *Epsilonproteobacteria* is recent, having been revealed by FISH and TEM observations that showed filamentous epibionts associated with the gill epithelia in *B. azoricus* (Assié et al., 2016). This recent discovery indicates that the microbial diversity associated with *Bathymodiolus* sp. may not yet be fully described. Indeed, Bathymodiolin mussels were mainly known to host dual symbioses with thiotrophic and methanotrophic *Gammaproteobacteria*-related symbionts, as described in multiple species (Duperron et al., 2009). After BLAST and phylogenetic analyses, our results indicate that some sequences of *O. dyvae* are related to both thiotrophic and methanotrophic symbionts of *B. azoricus* and some other sequences are more related to the *R. exoculata* epsilonproteobacterial symbiont. These results suggest that bacteria associated with *O. dyvae* may have the same metabolic capacities as the symbionts of *Bathymodiolus* or *Rimicaris* and play at least a partial role in the nutrition of their host. *O. dyvae* were sampled from the byssus of *Bathymodiolus* assemblages, which might be used as a stable point of attachment for the nematode. Thus, *B. azoricus* and *O. dyvae* shared the same habitat and some related lineages of *Epsilon*- or *Gammaproteobacteria*, but these were not entirely identical. Nematodes and mussels within this system might share the same microbial metabolic capacities, but harbor distinct bacterial communities; further investigations are needed to clarify whether OTU co-occurrences across the system are independent or not.

### Nematode-Prokaryote Interactions in Marine Environments

Most marine bacteria-nematode associations found to date involve only two sub-families: the *Stilbonematinae* and the *Astomonematinae*. The stilbonematines are long, thread-like nematodes that are abundant in carbonate sands on tropical coasts. Their symbionts are located in the thin layer of mucus produced by glandular sensory organs. The symbionts are chemolithoautotrophs, fuelled by chemical energy released during the oxidation of sulfur to produce organic compounds (Ott and Novak, 1989; Schiemer et al., 1990; Ott et al., 1991; Hentschel et al., 1999). The coastal stilbonematid nematode *Laxus oneistus* is capable of performing active nitrogen fixation (Petersen et al., 2017). The symbionts are transmitted vertically (Giere and Langheld, 1987), but symbiont phylogeny also reveals horizontal transmission events (Jannasch, 1995). The link between bacterial symbionts and the mucus secreted by *L. oneistus* suggests the potential conservation of host-microbe interaction mechanisms (Bulgheresi et al., 2006, 2011). Astomonematines are mouthless and lack an esophagus; they therefore depend entirely on their bacterial symbionts for nutrition. Similarly, to the stilbonematines, astomonematines are generally associated with reduced conditions, such as the subsurface intertidal layers of sulfur-enriched sediments (Ott et al., 1982) or sublittoral methane sources (Austen et al., 1993). *Astomonema* and *Parastomonema* are well-known in reducing environments, for example *Astomonema southwardorum* can be found in habitats such as pockmarks (Austen et al., 1993) and oxygen-poor sediments (Ott et al., 1982).

The first observation of Stilbonematines in the deep sea was reported by Van Gaever et al. (2006) in the anoxic micro-environments of Darwin Mounds (Northeast Atlantic Ocean). More recently, Stilbonematines were observed in deep-sea canyons along the continental margins of the West and Northeast Atlantic (Ingels et al., 2009, 2011a,b) and in Mediterranean pockmark fields (Zeppilli et al., 2012). Astomonematines have only recently been discovered in the deep-sea, [Northeast Atlantic Canyons (Ingels et al., 2009, 2011a,b)] and only one study suggests the existence of symbiosis in this group (Tchesunov et al., 2012). This latter study describes two species of nematode (*Parabostrichus bathyalis* and *A. southwardorum*) living in association with prokaryotic ectosymbionts (*Parabostrichus)* and endosymbionts (*Astomonema*) in two deep-sea systems: Gollum canals and Whittard canyon (Northeast Atlantic). The abundance of nematodes associated with chemotrophic ectosymbiotic (*Parabostrichus*) and endosymbiotic (*Astomonema*) bacteria suggests that chemosymbiotic fauna potentially play an important ecological role in these deep canyons and canals.

To our knowledge, our study is the first to provide strong evidence of a specific association between bacteria and nematodes from deep-sea hydrothermal vents. We can hypothesize some direct or indirect benefits of this epibiosis for the nematode such as its nutrition (by scraping and grazing on the hosts), detoxification such as for *Rimicaris* sp. (Zbinden et al., 2004; Jan et al., 2014) or prevention of pathogens. Without performing functional experiments, it is not possible to determine whether *O. dyvae* harbors any trophic symbionts, but the presence of a singular microbial epibiotic community together with the dominance of *Epsilonproteobacteria* and *Gammaproteobacteria* lineages opens the path to the study of unexplored prokaryote-eukaryote interactions in deep-sea hydrothermal vents. The discovery of a new well-adapted species of nematode able to reach high abundances in active hydrothermal areas, together with observations of close interactions of this nematode with prokaryotes, offers new insights on the links between different faunal compartments in vent ecosystems. Considering that hydrothermal vents may be particularly sensitive and vulnerable to human disturbance and threatened by the potential impacts of mineral resource extraction in the deep sea (seafloor massive sulfide deposits, Boschen et al., 2013), there is an urgent need to understand the ecology and biology of the species that thrive there, including neglected taxa such as nematodes.

## Author Contributions

LB, M-AC-B, and DZ analyzed the data and wrote the paper. LB carried out the molecular biology experiments, and phylogenetic and bioinformatics analyses. VC-G and LD performed FISH analysis. NG performed SEM analysis. All the authors read, edited, and approved the final manuscript.

## Conflict of Interest Statement

The authors declare that the research was conducted in the absence of any commercial or financial relationships that could be construed as a potential conflict of interest.

## Footnotes

1 https://dna.macrogen.com

2 http://www.geneious.com

3 www.mrdnalab.com

4 http://tree.bio.ed.ac.uk/software/tracer/

5 http://tree.bio.ed.ac.uk/software/figtree/
