# Supplementary material for: A Nematode of the Mid-Atlantic Ridge Hydrothermal Vents Harbors a Possible Symbiotic Relationship
Source: Front Microbiol. 2018 Sep 20;9:2246. doi: 10.3389/fmicb.2018.02246 (PMC6159746; doi:10.3389/fmicb.2018.02246)
Supplement: Supplementary file 1 [file Table_1.DOCX]

**Supplementary TableS1.** Number of reads (Nb) at different stages of processing in each *Oncholaimus dyvae* individual

| **Nematodes** | **Raw reads** | **Nb after quality filter** | **Final Nb (after affiliation)** |
| --- | --- | --- | --- |
| Od 2 | 46,465 | 40,727 | 32,42 |
| Od 3 | 83,22 | 70,467 | 56,922 |
| Od 4 | 52,032 | 42,980 | 35,962 |
| Od 5 | 58,168 | 52,368 | 41,134 |
| Od 6 | 65,119 | 57,203 | 34,212 |
| Od 7 | 101,066 | 85,537 | 71,135 |
| Od 12 | 57,184 | 49,268 | 40,76 |
| Od 13 | 67,731 | 59,358 | 49,115 |
| Od 14 | 39,896 | 34,320 | 25,131 |
| Od 15 | 82,249 | 69,232 | 58,831 |
| Od 16 | 75,274 | 64,252 | 49,798 |
| Od 17 | 39,67 | 34,569 | 27,363 |
| **Total** | **768,074** | **660,281** | **522,783** |

**Supplementary TableS2.** Taxonomies by nematode (Od)

| **Nematodes** | **Nb phyla** | **Nb classes** | **Nb orders** | **Nb families** | **Nb genera** | **Nb species** |
| --- | --- | --- | --- | --- | --- | --- |
| Od 2 | 12 | 20 | 39 | 65 | 93 | 107 |
| Od 3 | 12 | 20 | 38 | 67 | 101 | 110 |
| Od 4 | 11 | 19 | 35 | 54 | 78 | 83 |
| Od 5 | 12 | 20 | 35 | 65 | 105 | 115 |
| Od 6 | 13 | 23 | 44 | 81 | 124 | 138 |
| Od 7 | 10 | 18 | 36 | 68 | 104 | 112 |
| Od 12 | 10 | 18 | 33 | 58 | 88 | 96 |
| Od 13 | 12 | 22 | 36 | 59 | 90 | 99 |
| Od 14 | 13 | 24 | 44 | 77 | 109 | 122 |
| Od 15 | 10 | 17 | 32 | 59 | 86 | 95 |
| Od 16 | 10 | 18 | 34 | 59 | 93 | 107 |
| Od 17 | 9 | 17 | 34 | 63 | 94 | 105 |

Nb = number

**Supplementary TableS3.** Abundance (%) of four major phyla across all samples.

| **Nematodes** | **Proteobacteria** | **Bacteroidetes** | **Spirochaetae** | **Firmicutes** |
| --- | --- | --- | --- | --- |
| Od 2 | 31 | 2 | 8 | 44 |
| Od 3 | 54 | 37 | 4 | <1 |
| Od 4 | 17 | 51 | 29 | 1 |
| Od 5 | 27 | 22 | 10 | 22 |
| Od 6 | 63 | 3 | <1 | 12 |
| Od 7 | 34 | 26 | 31 | <1 |
| Od 12 | 53 | 13 | 23 | <1 |
| Od 13 | 15 | 3 | 51 | 7 |
| Od 14 | 20 | 26 | <1 | 25 |
| Od 15 | 19 | 54 | 6 | 4 |
| Od 16 | 16 | 27 | 4 | 1 |
| Od 17 | 51 | 10 | 7 | 11 |

**Supplementary TableS4.** Affiliation and relative abundance of eight common OTUs (that represent at least 1% of total reads) between all nematodes and extraction blank.

|  | **Affiliation (% similarity)** | **Blank Abundance** (% of total reads) | **Nematode Abundance** (% of total reads) |
| --- | --- | --- | --- |
| OTU 5 | *Actinobacteria Propionibacterium* (100%) | 13 | 5 |
| OTU 6 | *Firmicutes Staphylococcus* (100%) | 9 | 6 |
| OTU 17 | *Firmicutes Atopostipes* (98.6%) | 2 | 2 |
| OTU 12 | *Firmicutes Atopostipes* (99.7%) | 6 | 1 |
| OTU 15 | *Firmicutes Streptococcus* (100%) | 3 | 1 |
| OTU 8 | *Betaproteobacteria Delftia* (100%) | 6 | 3 |
| OTU 11 | *Gammaproteobacteria Pseudomonas* (100%) | 3 | 2 |
| OTU 14 | *Gammaproteobacteria Pseudomonas* (100%) | 4 | 2 |
