# Supplementary figures and images for: A Nematode of the Mid-Atlantic Ridge Hydrothermal Vents Harbors a Possible Symbiotic Relationship
Source: Front Microbiol. 2018 Sep 20;9:2246. doi: 10.3389/fmicb.2018.02246 (PMC6159746; doi:10.3389/fmicb.2018.02246)

Figure S1. Study area. Location of the Lucky Strike vent field on the Mid-Atlantic Ridge at 37°17.59 N, 32°169W.

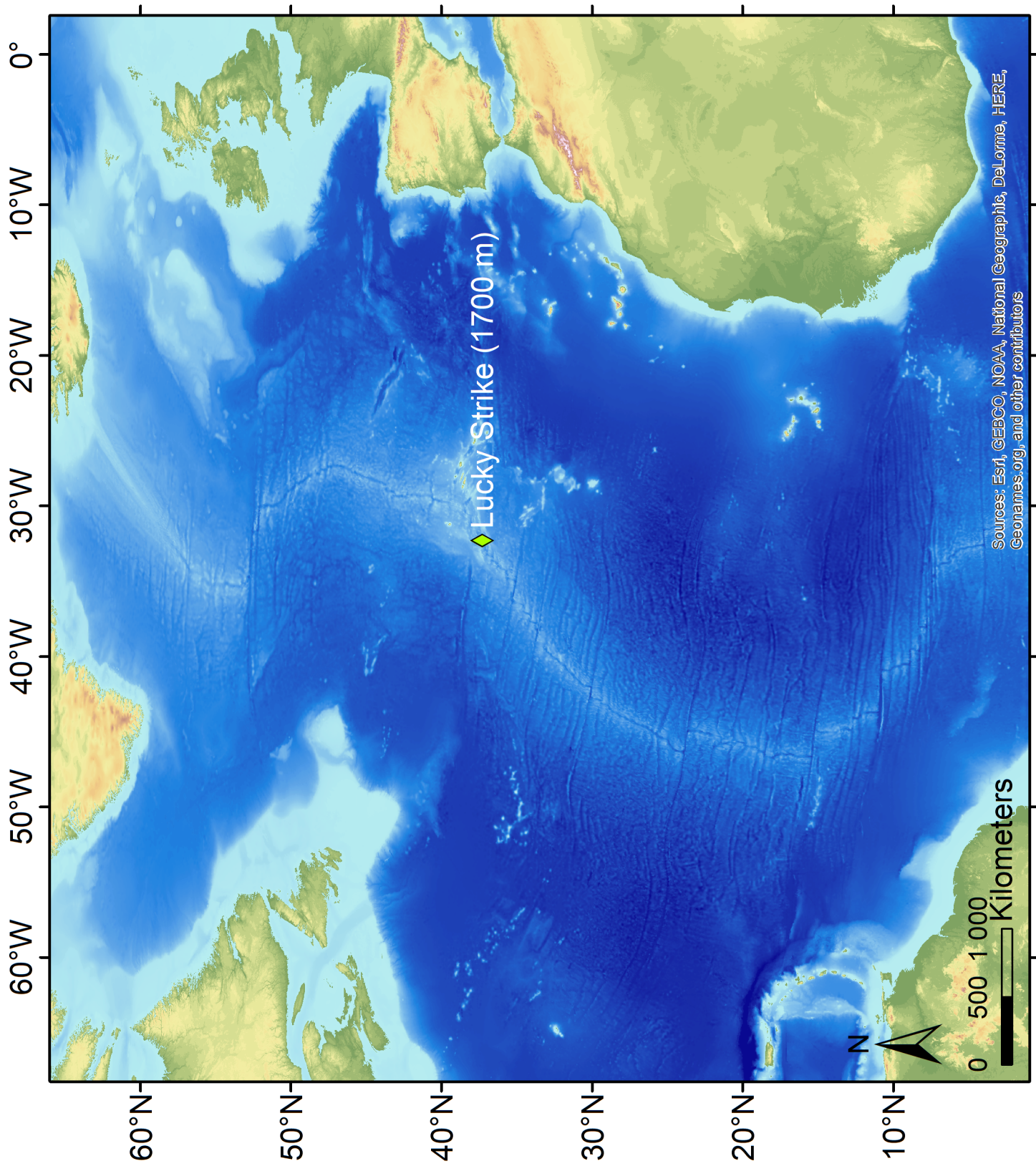

Supplement: Supplementary file 2 [file Image_1.PDF]

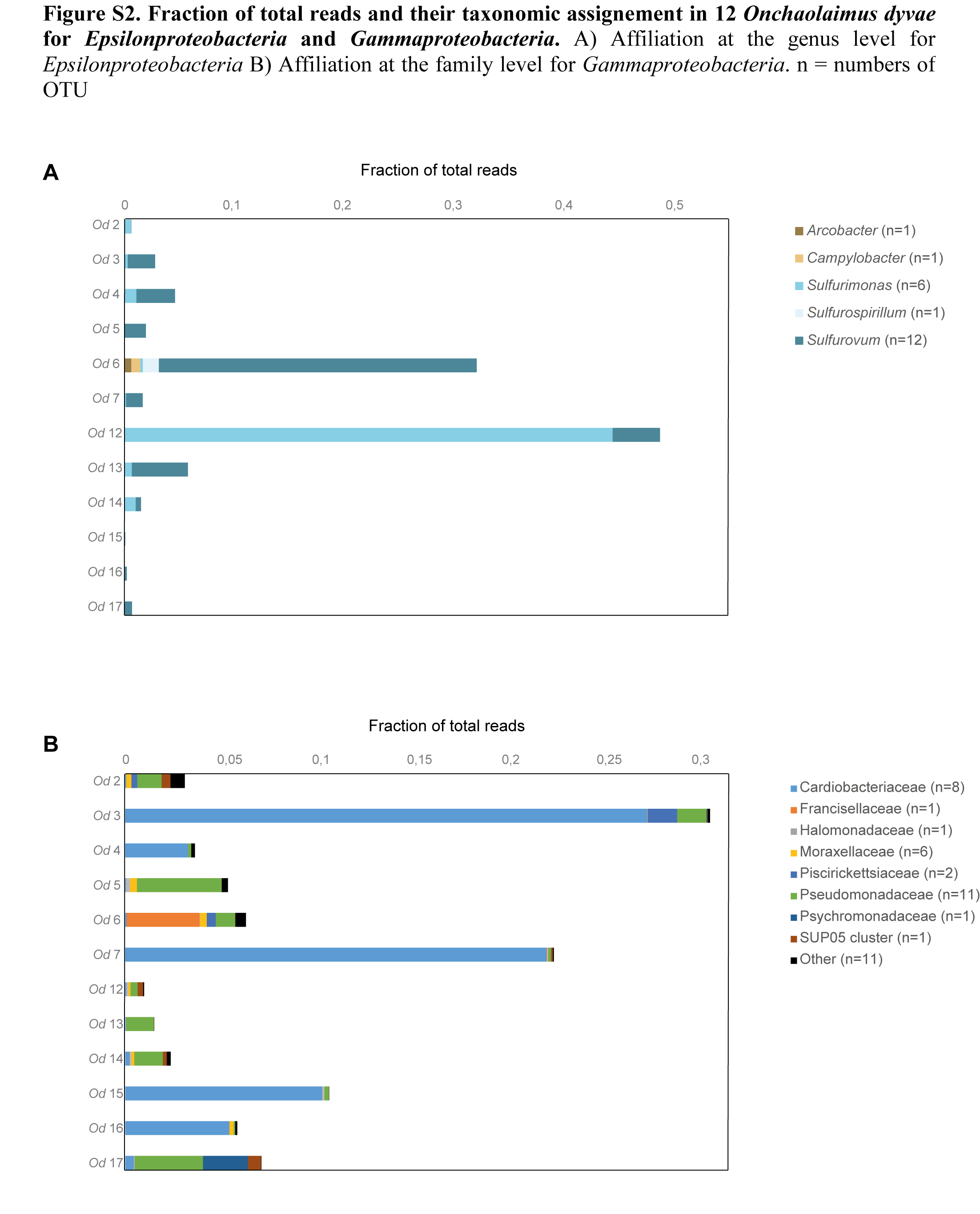

Supplement: Supplementary file 3 [file Image_2.TIF]
